# Relationship of Vitamin D-Deficient Diet and Irisin, and Their Impact on Energy Homeostasis in Rats

**DOI:** 10.3389/fphys.2020.00025

**Published:** 2020-01-31

**Authors:** Mahmoud Mustafa Ali Abulmeaty, Ali M. Almajwal, Iftikhar Alam, Suhail Razak, Mohamed F. ElSadek, Ghadeer S. Aljuraiban, Khulood S. Hussein, Asmaa M. Malash

**Affiliations:** ^1^Department of Community Health Sciences, College of Applied Medical Sciences, King Saud University, Riyadh, Saudi Arabia; ^2^Medical Physiology Department, Faculty of Medicine, Zagazig University, Zagazig, Egypt; ^3^Department of Physiology, Faculty of Medicine, King Abdulaziz University, Jeddah, Saudi Arabia; ^4^Department of Basic Medical Sciences, College of Medicine, Alfarabi Colleges, Riyadh, Saudi Arabia

**Keywords:** vitamin D deficiency, vitamin D, irisin, energy homeostasis, indirect calorimetry

## Abstract

**Background and Objective:**

Previous studies have identified the role of irisin and vitamin D in energy homeostasis. However, the effect of irisin and vitamin D on energy regulation has not been thoroughly investigated. Therefore, in this study, the effects of a vitamin D-deficient diet and irisin on total energy expenditure (TEE), food intake, and blood metabolites were investigated in rats.

**Methods:**

Sixteen healthy weaned male albino rats were randomly divided into two groups: a group fed a normal balanced growth diet (group A: *n* = 8) and a group fed a normocalcemic diet that is vitamin D deficient with limited ultraviolet (UV) light exposure (group B, *n* = 8). After 6 weeks, the volumes of respiratory gases were measured by open-circuit indirect calorimetry. Serum irisin, 25-OHVD_3_, calcium, insulin, and glucose levels were measured using ELISA. The respiratory quotient (RQ), energy expenditure, and Homeostatic Model Assessment for Insulin Resistance (HOMA-IR) were calculated.

**Results:**

Rats with hypovitaminosis D were hypoirisinemic. Food intake, RQ (to the range of using endogenous fat), and glucose levels reduced significantly, while insulin levels increased. Body weight and TEE were non-significant changed. Additionally, irisin was strongly and positively correlated with body weight under normal conditions (*r* = 0.905, *p* < 0.01), and a moderate negative correlation in group B (*r* = −0.429, *p* < 0.05). TEE and irisin showed no significant correlation.

**Conclusion:**

This study demonstrated that the early changes in energy homeostasis and irisin levels during states of hypovitaminosis D are affected by long-term consumption of a vitamin D-deficient diet with limited UV exposure.

## Introduction

Vitamin D insufficiency is highly prevalent (Dong et al., 2010; Wei and Giovannucci, 2010). Many reports documented the impact of vitamin D insufficiency in body weight regulation (Mithal et al., 2009; Farrell and Willis, 2012; Goshayeshi et al., 2012; Mai et al., 2012). However, it is unclear whether vitamin D insufficiency enhances weight gain or whether obesity modulates serum vitamin D levels. Studies aiming to regulate body weight by correcting vitamin D levels have yielded inconsistent findings (Zittermann et al., 2009; Salehpour et al., 2012), although other favorable effects, including reduced body fat weight (Salehpour et al., 2012), reduced inflammatory profile (Zittermann et al., 2009), and improved insulin resistance (Kamycheva et al., 2013) were observed.

In contrast to human trials, animal experiments have shown that vitamin D may play a role in weight gain. Vitamin D receptor knock-out mice were resistant to weight gain (Narvaez et al., 2009; Weber and Erben, 2013). Additionally, mice fed a vitamin D-deficient/insufficient diet were resistant to “western diets” (Bastie et al., 2012) and “high-fat diets” (Liu et al., 2013). Seldeen et al. (2017) reported that supplementing lean and obese mice with low cholecalciferol significantly reduced serum 25-OH vitamin D concentrations. Vitamin D insufficiency was not correlated to BMI or body fat (Seldeen et al., 2017). Findings on the effects of a vitamin D-deficient diet on weight gain and other parameters such as insulin, glucose levels, food intake have been inconsistent so far.

Irisin has been recently considered a potential candidate responsible for changes in weight and other related parameters in vitamin D deficiency. Irisin is a myokine – a peptide that causes browning of white fat, enhances burning of fat and, as a result, inhibits weight gain (Moreno-Navarrete et al., 2013). Irisin has been linked to the glucose/lipid metabolism (Huh et al., 2012; Choi et al., 2013; Moreno-Navarrete et al., 2013; Park et al., 2013; Stengel et al., 2013; Kurdiova et al., 2014; Sesti et al., 2014; Yan et al., 2014) and may have a preventive role in the adiposity development and the onset of diabetes (Lee et al., 2011; Moon, 2014). Irisin showed a stronger correlation to insulin resistance than other myokines; however, there is no consensus yet regarding the effectiveness of irisin secretion (Elsen et al., 2014).

Both irisin and vitamin D are important regulators of the musculoskeletal system and energy homeostasis. However, the effect of the irisin-vitamin D relationship on total energy expenditure (TEE), food intake, and substrate metabolism is well understood. We hypothesized that a vitamin D-deficient diet may lower serum irisin concentration and affect weight and TEE via changes in irisin. In the present study, we investigated (1) the effects of a vitamin D-deficient diet on the serum irisin concentration, and (2) whether a vitamin D-deficient diet associated with changes in body weight and TEE could be explained by variations in irisin levels using rat model.

## Materials and Methods

### Animals

This study was carried out using male Albino Wistar rats (*n* = 16). All experimental protocols were approved by the Animal Care and Ethics Committee in the College of Applied medical sciences, King Saud University (reference no: CAMS 52-35/36).

Rats were housed in a temperature-controlled room (21 ± 2°C) with 12-h light-dark cycles. All rats were fed a standard laboratory diet and had *ad libitum* access to tap water. After a 1-week acclimatization period, the male rats were randomly divided into two groups: group A, the normal control rats [vitamin D sufficient] (*n* = 8) and group B, the vitamin D-deficient rats (*n* = 8).

### Feeding Protocol

Immediately after weaning, the rats in group A were fed a normal balanced growth diet, the AIN-93G diet (Bio-Serv, United States) with 18% protein, 7% lipid, 60% carbohydrates, 5% fiber, 2.2% crude ash, in addition to 5.1 g calcium/kg, 2.8 g phosphorus/kg, and 1000 IU vitamin D/kg, and exposed freely to fluorescent lighting (60 cm away from the lamps). The rats in group B were fed a normocalcemic-vitamin D-deficient diet (Bio-Serv, United States). The rats also received limited ultraviolet exposure from fluorescent lights for 6 weeks by covering the upper surface of cages with opaque sheets and putting the cage in the lower level of the carrying track about 2 m away from the lamps. The nutrient composition of the vitamin D-deficient diet included 18% protein, 60% carbohydrates, 7% fat, 2.2% ash, and fibers. The micronutrients included 5.1 g/kg calcium, 2.8 g/kg phosphorus, and <50 IU/kg Vitamin D3. The animals in both groups had free access to tap water.

### Blood Sampling and Analysis

Blood samples (≈1 ml) from the lateral tail vein were used to assess the basal 25-hydroxyvitamin D3 (25OHVD), irisin, calcium, insulin, and glucose levels in both groups. Finally, all rats were euthanized and blood was sampled via cardiac puncture. Serum irisin, 25OHVD, calcium, glucose, and insulin were measured using ELISA kits according to the manufacturer protocol (MyBiosource, United States, Catalog numbers; MBS9356609, MBS261766, MBS283776, MBS7233226, and MBS724709, respectively).

Additionally, insulin resistance was assessed using the homeostatic model assessment (HOMA-IR) of β-cell function based on the method developed by Matthews et al. (1985) using the following formula [fasting insulin (U/l) × fasting glucose (mg/dl)/405]. A low HOMA-IR level indicates increased insulin sensitivity, and a high HOMA-IR level refers to low insulin sensitivity, i.e., insulin resistance (Matthews et al., 1985).

### Total Energy Expenditure Determination

After overnight fasting, rats from both groups were housed individually in Calo-cages with the TSE PhenoMaster system for 36 h (TSE, Germany), where volumes of respiratory gases were measured based on open-circuit indirect calorimetry. The respiratory quotient (RQ), an indicator of metabolic fuel, and total energy expenditure were calculated. Automatic food intake was also recorded with a precision of 0.01 g through a calibrated sensor. Measurements were taken every 15 min, and those of the first 6 h corresponding to the acclimatization period were deleted.

The parameters used for analysis were volume of respiratory oxygen and carbon dioxide (VO_2_ and VCO_2_) per hour per kg of body weight, per hour per kg lean body mass, and per hour only. Accordingly, respiratory quotient (RQ) and total energy expenditure (TEE) were calculated. The TEE was presented as calories per hour per kg of body weight or lean body mass and as calories per hour. The lean body mass was estimated to be 75% of the body weight (Ali Abulmeaty et al., 2019).

### Statistical Analysis

SPSS software (ver.24.0, SPSS, Chicago, IL, United States) was used for all statistical analyses. All study variables were tested for normality by using the Kolmogorov–Smirnov and Shapiro–Wilk tests. All variables followed normal distribution, as the *p*-values were >0.05, and the data were expressed as means ± standard deviation (SD). An independent sample *t*-test was performed to observe differences between the groups. The paired sample *t*-test was also used to compare basal and final measurements. The Spearman correlation coefficient test was used for testing correlation among all parameters. *P*-values < 0.05 indicated statistical significance.

## Results

### General Characteristics

The baseline characteristics of the study animals in comparison to their final conditions are shown in Table 1. All rats (*n* = 16) were similar with respect to age, body weight, and other basal parameters.

**TABLE 1:** Baseline characteristics of the study groups vs. final measurements.

Parameters	Group A (Vitamin D sufficient) (*n* = 8)		*P*-value	Group B (Vitamin D deficient) (*n* = 8)		*P*-value
	Basal	Final		Basal	Final	
Age (weeks)	4.57 ± 0.43	11.57 ± 0.43	0.000	4.43 ± 0.43	11.43 ± 0.43	0.000
Weight (g)	70.31 ± 5.54	261.28 ± 31.25	0.000	68.09 ± 6.65	270.45 ± 26.81	0.000
Irisin (ng/ml)	296.68 ± 58.78	424.42 ± 4.90	0.000	282.96 ± 22.10	383.55 ± 30.42	0.000
25OHVD (ng/ml)	29.75 ± 3.03	36.84 ± 7.74	0.054	30.07 ± 2.82	15.88 ± 4.88	0.000
Ca (mg/dl)	9.78 ± 1.06	10.23 ± 0.79	0.338	10.16 ± 0.98	7.11 ± 0.98	0.001
Insulin (mIU/l)	7.54 ± 0.75	8.41 ± 0.38	0.034	7.99 ± 1.01	11.48 ± 1.55	0.000
Glucose (mg/dl)	83.26 ± 10.81	87.61 ± 3.41	0.288	79.96 ± 9.77	58.64 ± 3.58	0.000
HOMA-IR	1.55 ± 0.28	1.82 ± 0.10	0.026	1.57 ± 0.26	1.65 ± 0.13	0.452

### Effects of a Normocalcemic-Vitamin D-Deficient Diet on Irisin Levels

**FIGURE 1:**
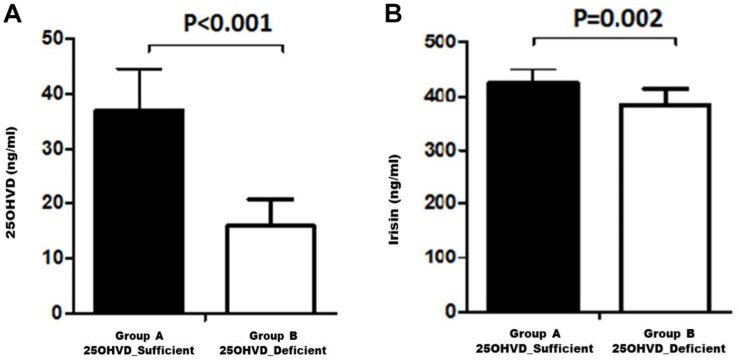
Serum 25OHVD **(A)** and irisin levels **(B)** in the study groups.

Figure 1 shows a comparison of the irisin concentration in rats in group A (vitamin D sufficient) and group B (vitamin D deficient). Irisin levels were significantly higher in group A compared to group B.

Table 2 presents the study parameters of groups A and B. The body weight did not differ between the two groups (*p* = 0.54). Similarly, the mean TEE in group A was not statistically different to that in group B (5.18 ± 0.80 vs. 5.94 ± 1.05 Kcal/h/Kg, *p* = 0.13). There was a significant reduction in RQ in group B in favor of more lipid utilization, combined with a significant increase in insulin level, and decrease in food intake, and HOMA-IR indicator compared to the corresponding values in group A.

**TABLE 2:** Comparison of the measured parameters between vitamin D-sufficient and -deficient rats.

Parameters	Group A (Vitamin D sufficient) (n=8)	Group B (Vitamin D deficient) (n=8)	*P*-value
	Mean ± SD	Mean ± SD	
Ca (mg/dl)	10.23 ± 0.79	7.11 ± 0.98	0.000
VO_2_ (ml/h/kg)	1203.30 ± 192.75	1076.93 ± 159.94	0.175
VO_2_ (ml/h/kg lean body mass)	890.54 ± 142.68	797.01 ± 118.36	0.176
VO_2_ (ml/h)	360.99 ± 57.84	323.07 ± 48.00	0.176
VCO_2_ (ml/h/kg)	1078.41 ± 271.38	841.56 ± 161.71	0.052
VCO_2_ (ml/h/kg lean body mass)	798.08 ± 200.85	622.82 ± 119.69	0.052
VCO_2_ (ml/h)	323.52 ± 81.43	252.45 ± 48.50	0.052
Respiratory Quotient	0.89 ± 0.10	0.78 ± 0.05	0.020
TEE (kcal/h/kg)	5.94 ± 1.05	5.18 ± 0.80	0.126
TEE (kcal/h/kg lean body mass)	4.39 ± 0.78	3.83 ± 0.59	0.125
TEE (kcal/h)	1.78 ± 0.31	1.55 ± 0.24	0.126
Weight (g)	261.28 ± 31.25	270.45 ± 26.81	0.539
Food intake (g/day)	9.73 ± 3.35	6.49 ± 1.88	0.031
Insulin (mIU/l)	8.41 ± 0.38	11.48 ± 1.55	0.000
Glucose (mg/dl)	87.61 ± 3.41	58.64 ± 3.58	0.000
HOMA-IR	1.82 ± 0.10	1.65 ± 0.13	0.012

### Correlations of Irisin and 25-OHVD_3_

**FIGURE 2:**
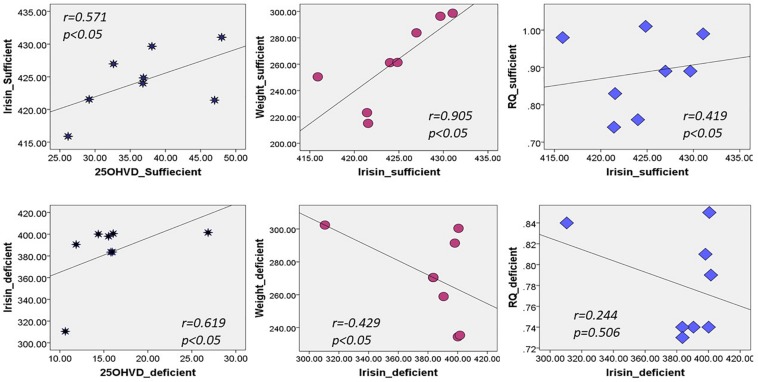
Correlation between irisin and 25OHVD, body weight, and RQ in both study groups.

Figure 2 shows that irisin was positively correlated with 25OHVD in both groups (*n* = 8, *r* = 0.571 and 0.619, respectively, *p* < 0.05). Additionally, irisin was significantly correlated with body weight in group A (*r* = 0.905, *p* < 0.05), and negatively in group B (*r* = −0.429, *p* < 0.05). Irisin was also correlated with RQ in the normal control group rather than in the vitamin D-deficient group. The 25OHVD_3_ showed a significant correlation with insulin level, HOMA-IR, and calcium concentration in group A (Table 3).

**TABLE 3:** Spearman correlation coefficients of the measured parameters with Irisin and 25-OHD_3_.

Parameters	Group A (Vitamin D sufficient)		Group B (Vitamin D deficient)	
	Irisin	25OHVD_3_	Irisin	25OHVD_3_
Irisin	1	0.571*	1	0.619*
VO_2_ by body weight	–0.143	0.071	–0.167	0.024
VO_2_ by lean body mass	–0.143	0.071	–0.167	0.024
VO_2_ by cage	–0.143	0.071	–0.167	0.024
VCO_2_ by body weight	–0.048	0.000	–0.048	0.048
VCO_2_ by lean body mass	–0.048	0.000	–0.048	0.048
VCO_2_ by cage	–0.048	0.000	–0.048	0.048
Respiratory quotient	0.419*	–0.036	0.244	0.000
TEE by body weight	–0.143	0.071	–0.119	–0.024
TEE by lean body mass	–0.143	0.071	–0.119	–0.024
TEE by cage	–0.143	0.071	–0.119	–0.024
Weight	0.905**	0.238	−0.429*	0.000
Food intake	0.214	0.024	0.048	–0.190
Insulin	0.072	0.802*	–0.180	–0.108
Glucose	0.071	–0.190	–0.024	–0.167
HOMA-IR	0.335*	0.563*	−0.431*	–0.216
Calcium	0.252	0.503*	–0.048	0.238

*Correlation is significant at the 0.05 level (2-tailed), **Correlation is significant at the 0.01 level (2-tailed).

## Discussion

In this experiment, we demonstrated that vitamin D deficiency may affect weight and TEE via irisin. The vitamin D-deficient rats in group B had lower irisin levels than rats in group A (*p* = 0.02; Figure 1), consistent with our hypothesis. Interestingly, there was a significant reduction in RQ in group B, along with a significant decrease in food intake, glucose levels, and HOMA-IR, in addition to a significant increase in insulin level compared to those in group A. Furthermore, irisin was positively correlated with HOMA-IR and body weight in the group A, while the correlation was inversed in the vitamin D-deficient group. Additionally, irisin was significantly and positively correlated with RQ in the vitamin D-sufficient but not in the vitamin D-deficient group, whereas 25OHVD_3_ was significantly correlated with insulin level, HOMA-IR, and calcium concentration in group A.

In our experiment, serum irisin was directly correlated with HOMA-IR, body weight, and RQ in the vitamin D-sufficient group, whereas it was negatively correlated with HOMA-IR and body weight in the vitamin D-deficient group. Park et al. (2013); Ebert et al. (2014), and few others (Liu et al., 2013; Chen et al., 2015) found similar results of significant positive correlations between irisin and HOMA-IR. In contrast, Al-Daghri et al. (2014) found that irisin was inversely correlated with HOMA-IR in women, whereas Moreno-Navarrete et al. (2013) reported inverse correlations in obese men. A recent, large-scale exploratory study, including 1,115 obese Chinese men and women, showed that those with high irisin levels had lower blood glucose levels or metabolic syndrome compared to controls (Yan et al., 2014). The inconsistent findings across studies and between genders (Moreno-Navarrete et al., 2013; Al-Daghri et al., 2014), suggest that the irisin level is affected by fat distribution, insulin sensitivity, and the level of vitamin D, which was not previously investigated. Animal studies may explain this mechanism, as overexpression of the irisin precursor, fibronectin-type III domain-containing 5 (FNDC5), in obese mice increased TEE and insulin sensitivity, and decreased hyperglycemia, hyperlipidemia, and hypertension (Xiong et al., 2015). Since FNDC5 mRNA was also detected in adipose tissue and skeletal muscle (Seldeen et al., 2017), higher levels of irisin increased thermogenesis and TEE in high fat fed mice (Bostrom et al., 2012). Muscle cells treated with irisin enhanced the uptake of glucose and fatty acid (Perakakis et al., 2017). Irisin also increased GLUT4 and PPARα gene expression, which modulate glycogenolysis and gluconeogenesis, respectively (Perakakis et al., 2017). However, in patients with diabetes or obesity, irisin levels decreased due to a drop in FNDC5 expression triggered by chronic hyperglycemia and hyperlipidemia (Kurdiova et al., 2014). Furthermore, irisin injections stimulated browning of subcutaneous fat, suggesting that irisin may have therapeutic effects, however, these findings need to be confirmed. Thus, it was hypothesized that a fat-derived feedback mechanism in obese individuals lead to increased production of irisin; the secreted irisin into blood ameliorates insulin resistance by increasing the expression of uncoupling protein-1 gene, resulting in the browning of white fat (Sanchis-Gomar and Perez-Quilis, 2014). Future studies are needed to confirm this proposed mechanism.

We also demonstrated that irisin and vitamin D were directly correlated in both groups. This was in line with findings of a one-year-long intervention study on male and female human subjects fed a vitamin D-rich diet and exposed to sunlight showed that levels of irisin only significantly increased in males compared to those in the controls (Al-Daghri et al., 2014). These results may be attributed to pancreatic β-cells which play a role in the pathogenesis of diabetes through variations in genes controlling metabolism and expression of the receptors (Giri et al., 2017). This expression may lead to an activation of PGC1α, a transcriptional coactivator that increases FNDC5 mRNA expression, resulting in high irisin secretion into the blood. *In vivo*, the parathyroid hormone played a role in regulating the expression of FNDC5/Irisin, which was directly related to changes in serum vitamin D and calcium (Palermo et al., 2019), however, these findings need to be further confirmed in future studies.

The main strengths of the study include the use of the animal model which allowed us to examine interrelationships between serum vitamin D insufficiency and irisin, independent of lifestyle or genetic factors presented in human studies. However, since animal models use of receptor knockouts or dietary removal and/or manipulation to simulate vitamin D-deficient conditions, this may not accurately simulate the more prevalent condition of insufficiency in humans. There are some limitations to this study: First, we could not measure blood parameters at different time points, thus we were unable to investigate variations in irisin levels. Second, due to the small sample size, we were unable to further divided the animals into groups in order to assess the effects of different vitamin D concentrations in the diet.

## Conclusion

This study demonstrated that the early changes in energy homeostasis and irisin levels during states of hypovitaminosis D are affected by long-term consumption of a vitamin D-deficient diet. Further research is needed to identify the molecular basis of these findings. Our findings also suggest that both the normal balanced growth diet and the normocalcemic-vitamin D-deficient diet with limited ultraviolet light exposure for 6 weeks failed to modulate body weight and TEE. A 6-week vitamin D-deficient diet induced a vitamin D insufficiency in rats (serum 25-OHVD_3_ levels: <20 ng/ml) combined with significant changes in serum levels of irisin, Ca, insulin, and glucose.

## Data Availability Statement

The raw data supporting the conclusions of this article will be made available by the authors, without undue reservation, to any qualified researcher.

## Ethics Statement

The animal study was reviewed and approved by Ethics Committee in the College of Applied Medical Sciences, King Saud University.

## Author Contributions

MA contributed to the practical work, indirect calorimetry, and the writing of the manuscript. AA contributed to the study design and indirect calorimetry. IA contributed to the lab work and writing of manuscript. SR contributed to the lab work. ME contributed to the statistical analysis. GA contributed to the preparation of figures and tables and the writing of the results and discussion. KH contributed to the analysis of indirect calorimetry results. AM contributed to the administrative work.

## Conflict of Interest

The authors declare that the research was conducted in the absence of any commercial or financial relationships that could be construed as a potential conflict of interest.
